# Cognitive impairment without altered levels of cerebrospinal fluid biomarkers in patients with encephalitis caused by varicella-zoster virus: a pilot study

**DOI:** 10.1038/s41598-020-79800-2

**Published:** 2020-12-28

**Authors:** Marie Eckerström, Staffan Nilsson, Henrik Zetterberg, Kaj Blennow, Anna Grahn

**Affiliations:** 1 Sahlgrenska University Hospital Memory Clinic, Mölndal, Sweden; 2 Department of Psychiatry and Neurochemistry, Institute of Neuroscience and Physiology, The Sahlgrenska Academy at University of Gothenburg, Mölndal, Sweden; 3 Department of Mathematical Statistics, Chalmers University of Technology, Gothenburg, Sweden; 4 Clinical Neurochemistry Laboratory, Sahlgrenska University Hospital, Mölndal, Sweden; 5 Department of Neurodegenerative Disease, UCL Institute of Neurology, Queen Square, London, UK; 6 UK Dementia Research Institute, London, UK; 7 Department of Infectious Diseases, Institute of Biomedicine, The Sahlgrenska Academy at University of Gothenburg, Gothenburg, Sweden; 8 Department of Infection, Sahlgrenska University Hospital, Gothenburg, Sweden

**Keywords:** Alzheimer's disease, Viral infection

## Abstract

Varicella-zoster virus (VZV) is one of the most common agents causing viral infections of the central nervous system (CNS). VZV encephalitis is associated with severe neurological sequelae, despite antiviral treatment. Cognitive impairment has been reported and VZV has been associated with dementia. Our aim was to investigate the cognitive impairment and cerebrospinal fluid biomarkers in a follow-up study of patients with VZV encephalitis. Thirteen patients with VZV encephalitis, diagnosed by detection of VZV DNA in cerebrospinal fluid (CSF) by PCR and concomitant symptoms of encephalitis, were included. Neuropsychological assessment in parallel with a lumbar puncture to obtain CSF was performed 1.5–7 years after acute disease. The CSF biomarkers neurofilament light chain (NFL), S100B, glial fibrillary acidic protein (GFAP), amyloid-β (Aβ) 40 and Aβ42, total tau (t-tau) and phosphorylated tau (p-tau) were analysed and compared to controls (n = 24). Cognitive impairment was shown in the domains of executive functions and speed/attention and to a minor degree in the domains of learning/memory and language, indicated by a significantly poorer performance on seven neuropsychological test variables. No convincing evidence of alterations in concentrations of biomarkers in the CSF were shown. Our results indicate that patients with VZV encephalitis suffer from cognitive impairment long time after acute disease. Importantly, these impairments do not seem to be accompanied by biomarker evidence of ongoing neuronal or astrocytic injury/activation or induction of dementia-related brain pathologies by the infection.

## Introduction

Varicella-zoster virus (VZV) is one of the most common viral agents causing central nervous system (CNS) infections in the Western world. The neurological complications caused by VZV include a wide spectrum of manifestations, such as meningitis, encephalitis, facial paralysis, myelitis, cerebellitis and stroke-like syndromes^1^, and may occur after both primary (varicella) and reactivated (herpes zoster) VZV infection. The typical rash of herpes zoster is lacking in up to 40% of patients with reactivated VZV and concomitant neurological complications, which may hamper the diagnosis^1^. The neurological sequelae of VZV CNS infections are reported to be of varying severity but are most pronounced in patients with encephalitis and include both motor and cognitive dysfunctions^1−4^. In a French follow-up study of 15 patients with VZV encephalitis, up to half of the patients were reported with moderate disability but independent in daily life 3 years after acute disease^3^. The cognitive sequelae are so far unexplored. Only few follow-up studies comprising neuropsychological assessments are available^5–7^. In one of these, cognitive impairment was shown 3 years after acute disease compared to controls and included dysfunctions in the domains of *speed and attention*, *executive functions* and *learning and memory*^7^. In addition, herpes zoster has been associated with dementia in recent epidemiological studies^8,9^. In a 5-year follow-up study, patients with herpes zoster ophthalmicus had a threefold greater risk to develop dementia, most commonly of the Alzheimer type, compared to healthy individuals^9^.

One method of identification of brain pathophysiology after CNS infections is by measuring cerebrospinal fluid (CSF) concentrations of various biomarkers. Neurofilament light chain (NFL) which is a marker of neuronal damage, is shown to be increased several months after onset of disease in patients with VZV CNS infections, most pronounced in patients with encephalitis^10^. Changes in concentrations of S-100B and glial fibrillary acidic protein (GFAP) also indicate astroglial cell leakage in these patients^10,11^. The Alzheimer’s disease (AD) CSF biomarkers amyloid-β (Aβ) 40 and Aβ42, reflecting Aβ metabolism and amyloid plaques^12^, have recently been investigated in an in-vitro study which suggests that VZV infection may increase the toxic amyloid burden and contribute to amyloid-associated disease progression^13^. Aβ40 and Aβ42 are generally considered markers of plaque pathology in AD patients but Aβ42 has also been shown to be a marker of neuroinflammation in CNS infections including herpes simplex encephalitis (HSE)^14,15^. CSF total tau (t-tau) and phosphorylated tau (p-tau) are other biomarkers of AD that reflect cortical axonal degeneration and neurofibrillary pathology, respectively^12^, and both have shown to be increased in HSE^15^.

In addition, some of these CSF biomarkers have been demonstrated to correlate with the results of neuropsychological testing. Levels of CSF Aβ42, t-tau and p-tau have been associated with cognitive performance in patients with AD and mild cognitive impairment^16,17^ and levels of NFL in CSF have been shown to correlate to cognitive performance in patients with multiple sclerosis^18^.

Altogether, the knowledge of neurological sequelae in patients with VZV CNS infections, including cognitive impairment, is very limited. This disease needs to be further explored to better understand the neuropathogenesis and also the prognosis and the need of rehabilitation for these patients.

Therefore, our aim was to prospectively investigate the cognitive impairments in patients with previous VZV encephalitis in a long-term follow-up study and to relate the results to differences in CSF concentrations of biomarkers indicating CNS pathology (NFL, GFAP, S-100B, Aβ40, Aβ42, t-tau and p-tau), measured in parallel with the neuropsychological assessments > 1.5 years after acute disease.

## Methods

### Patients and controls

Patients admitted to a hospital in the region of Västra Götaland (population 1.7 million), Sweden, between 2007–2016, were enrolled in this follow-up study. The enrolled patients had VZV DNA in their CSF as detected by real-time PCR and had contemporary neurological symptoms diagnosed as encephalitis. The encephalitis diagnosis was based on previous published criteria^10^ and was defined as acute signs of parenchymatous brain dysfunction in addition to two of the following symptoms: fever > 38 °C, pleocytosis with leukocytes > 4 × 10^6^/L or electroencephalogram (EEG) abnormalities. At the time of acute disease most patients underwent magnetic resonance imaging (MRI) or computed tomography (CT) of the brain within the first 10 days. Antiviral treatment was in most cases given according to national recommendations in Sweden (Health Care Program for viral CNS infections, 2016), i.e. in encephalitis, 10–15 mg/kg t.i.d of acyclovir is given for 7–14 days. Clinical data are presented in Table 1.

**Table 1:** Demographic data of 13 patients with previous encephalitis caused by reactivated varicella-zoster virus.

Gender/age at follow-up	Education (years)	Viral load at acute disease (copies/ml)	MRI/CT at acute disease	Iv treat-ment (days)	Follow-up (mo. after symptom onset)	LP/cognitive testing at follow-up
F/82	7	100	MRI ND/CT neg	4	85	ND/Yes
F/63	4,5	1,600,000	MRI and CT neg	7	53	Yes/Yes^c^
M/28	12	25,000	MRI and CT neg	8	42	Yes/Yes
F/24	12	400	MRI ND/CT neg	14	41	Yes/Yes
M/85	7	6,300	Wide-spread white matter changes and general atrophy	^a^	18	Yes/Yes
M/76	7	7,100	Wide-spread white matter changes periventricularly and subcortically and spot-like changes in the basal ganglia	7	91	Yes/Yes
M/52	11	20,000	Left-sided thalamic and occipital ischemic changes	13	91	Yes/Yes
F/34	13	25 million	ND	12	39	Yes/Yes
M/74	10	500,000	ND	6	33	Yes/Yes
F/35	16	12,600	MRI ND/CT neg	10	36	Yes/Yes
M/32	15	31,600	MRI and CT neg	10	50	Yes/Yes
M/58	5	ND^b^	MRI and CT neg	14	20	Yes/Yes^c^
M/73	ND	316,000	MRI neg/CT ND	11	19	Yes/ND

*CT* computer tomography, *Iv* intravenous, *ND* not done, *LP* lumbar puncture, *MRI* magnetic resonance imaging.

^a^Oral treatment with valacyklovir 1 g × 3, 7 days.

^b^Only qualitative PCR analysis was performed.

^c^Limited Swedish language.

One and a half to 7 years after acute disease caused by reactivated VZV, the patients were invited to participate in a follow-up study including neuropsychological testing and lumbar puncture for analysis of CSF biomarkers. At the time of testing they were asked about functional and cognitive impairment, current medication and alcohol habits. The exclusion criteria for patients were other concomitant CNS disease and other somatic diseases that made testing impossible.

Thirteen patients consented to participate in the study. Twelve out of 13 patients had a lumbar puncture, within a month before or after the neuropsychological testing. In the thirteenth patient, an 82-year-old woman with back problems, the lumbar puncture did not succeed. Twelve out of 13 patients underwent the neuropsychological tests at a median of 41.5 months (range: 19–85) after the acute disease of VZV encephalitis. The patient who was not tested, a 73-year-old man, did not come for the testing because of a misunderstanding. Of the 12 patients who underwent neuropsychological testing, another two patients had limited Swedish language proficiency, which made it difficult to interpret their test results. They were excluded from the statistical analysis. Of these 10 patients, who were finally analysed, all had a lumbar puncture. For clinical data, see Table 1.

One patient was immunocompromised, suffering from systemic lupus erythematosus (SLE) and was receiving mycophenolic acid. During the time of testing, one patient received medication for pain with tramadol. Otherwise, no patients were on any medication affecting the cognitive test results. All patients that were tested declared very modest alcohol consumption (maximum 2–3 glasses of wine/week).

At follow-up, two patients were legally disabled, in one case regarded as a consequence of the previous VZV CNS infection. Six out of 13 patients were retired at normal age. Three patients had returned to work, one patient was enrolled as a student and one patient was unemployed. At follow-up, one patient had balance disorders and a hearing deficit regarded as neurological sequelae of the VZV CNS infection, which disabled him in daily life. The other patients had no other notable neurological deficits besides the cognitive impairments.

CSF samples from 24 non-infectious subjects, matched for age and gender, that had sought care for headaches were included as controls. Examination of all control subjects revealed normal neurological status, normal CSF cell count, normal CSF albumin concentration and no clinical signs of herpes zoster.

### CSF analysis

At the time of acute disease, non-centrifuged CSF was analysed for cell counts and VZV DNA by a quantitative in-house PCR with a sensitivity of 100 copies/ml of VZV DNA^1^. At follow-up 1.5 to 7 years after the acute infection, non-centrifuged CSF was analysed for cell counts. After centrifugation, supernatants were aliquoted and stored at –70 °C pending biochemical analyses. All CSF samples were then analysed on one occasion using one batch of reagents for the following biomarkers: GFAP, NFL, S-100B, Aβ40, Aβ42, t-tau and p-tau. The concentrations of GFAP and NFL were determined using previously described in-house ELISA methods, shown to specifically react with GFAP and NFL in CSF, and having analytical sensitivity high enough to measure these proteins in any CSF samples, also from healthy people^19,20^. Detection levels for the NFL and GFAP in-house ELISA methods are reported to 78 ng/l and 16 ng/l respectively^19,20^. CSF levels of S-100B were determined using the cobas Elecsys system and the S100B reagent kit (Roche Diagnostics, Basel, Switzerland). Aβ40, Aβ42, t-tau and p-tau concentrations were measured using Lumipulse G β-amyloid 1–42 (no. 230336), β-amyloid 1–40 (no. 231524), total Tau (no. 230312) and pTau 181 (no. 230350) assays on the fully automated LUMIPULSE G600II instrument according to instructions from the manufacturer (Fujirebio, Ghent, Belgium). The limit of detection for Lumipulse G total Tau and pTau 181 are 141 ng/l and 0.28 ng/l, respectively, according to manufacturer (Fujirebio, Ghent, Belgium). The coefficients of variation for all biochemical analyses were below 10%.

### Neuropsychological testing and procedure

A neuropsychological test battery including 11 tests and comprised of 18 test variables was used to capture cognitive impairments from different cognitive domains. The battery comprises the domains of *speed and attention* (symbol digit modalities test (SDMT); trail making test A (TMT A); continuous performance test (CPT-II) reaction time), *learning and episodic memory* (California verbal learning test (CVLT); brief visuospatial memory test revised (BVMT-R), *visuospatial functions* (block tapping test; the silhouettes subtest from the visual object and space perception battery), *language* (Boston naming test (BNT); category fluency animal naming) and *executive functions* (continuous performance test (CPT-II) omissions and commissions; trail making test B (TMT B); letter-number sequencing). The neuropsychological tests are further described in Table 2. The tests were administered by psychologists in training, supervised by a specialized neuropsychologist. All tests were administered in a standardised sequence designed to reduce the risk of contamination in the memory tests. The patients’ test results were analysed in relation to previously published age- and education stratified normal data, see Table 2 for references.

**Table 2:** Neuropsychological tests, domains, functions and source of normal data.

Cognitive domain	Neuropsychological test	Task and function	Specific variables analysed	References
Executive functions	Continuous performance test (CPT) II	Computerized target response task. Involves several aspects of sustained and selective attention, executive control, and reaction time	Omissions (failure to respond to target); commissions (response to non-target); reaction time (hit RT—average speed of correct responses)	^32^
	Letter-number-sequencing	Recall a series of numbers in increasing order and letters in alphabetical order. Involves working memory, attention, executive control	Sum of correct responses	^33^
	Trailmaking test B	Timed sequencing task, alternating letters and numbers. Visual scanning, psychomotor speed, cognitive flexibility	Time in seconds	^34^
Learning and memory	California verbal learning test (CVLT)	Word list learning (trial 1–5) and recall. Involves verbal episodic learning and memory	Total learning (sum of correct responses in trial 1–5); immediate recall; delayed recall	^35^
	Brief visuospatial memory test revised (BVMT-R)	Timed spatial figure learning (trial 1–3) and recall. Involves spatial episodic learning and memory	Learning (trial 1–3); delayed recall	^36^
Speed and attention	Trailmaking test A	Timed sequencing task, numbers. Visual scanning and psychomotor speed	Time in seconds	^34^
	Symbol digit modalities test	Timed substitution task using a reference key. Involves attention, psychomotor speed, perceptual speed, visual scanning	Sum of correct responses	^37^
	Continuous performance test (CPT) II	See above	Reaction time (hit RT—average speed of correct responses)	^32^
Language	Boston naming test	Picture naming task. Involves confrontational word retrieval	Sum of correct responses	^38^
	Category fluency (animals)	Timed word generation within a semantic category. Involves verbal fluency and executive control	Sum of correct responses	^39^
Visuospatial functions	Block tapping test	Tapping spatially separated blocks in predefined sequences of increasing length. Involves visuospatial working memory	Sum of correct responses	^33^
	Silhouettes (VOSP battery)	Picture task, identifying objects and animals presented as silhouettes. Involves visuospatial perception	Sum of correct responses	^40^

### Statistical analysis

Differences in biomarker log concentrations between patients and matched controls were analysed with linear mixed models. The patients’ cognitive test results were transformed into T-scores using previously published age- and education stratified normal data (see Table 2) and directed so that a better performance equals a higher T-score. Differences between T-scores and norm values (i.e. mean 50 and standard deviation 10) were analysed by one-tailed one-sample t-tests. Association between age and T-scores were analysed with Pearson correlation tests. Spearman’s rank test was used for correlations between biomarker concentrations and T-scores.

## Results

### CSF biomarkers

**Figure 1:**
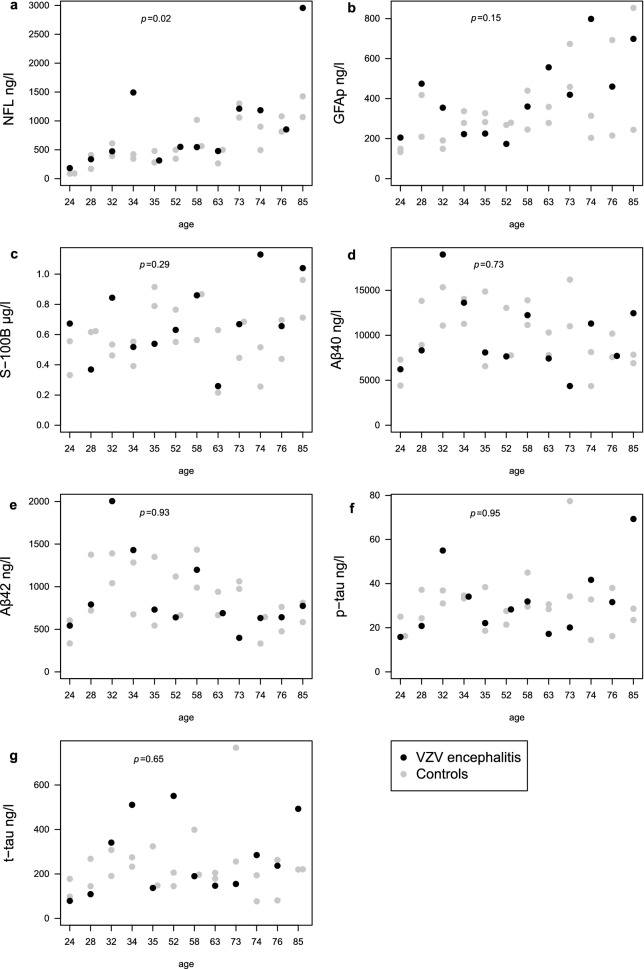
(**a–e**) Concentrations of cerebrospinal fluid biomarkers in patients with encephalitis caused by reactivated varicella-zoster virus (n = 12), 41 months (range: 19–85) after acute disease, and their controls (n = 24) (**a**) neurofilament light chain (NFL), (**b**) glial fibrillary acidic protein (GFAP), (**c**) S-100B, (**d**) amyloid-β 40 (Aβ40), (**e**) amyloid-β 42 (Aβ42), f) phosphorylated tau (p-tau), g) total tau (t-tau).

Significantly increased concentrations of NFL, indicating neuronal damage, were shown in the patients with VZV encephalitis (*n* $=$ 12) (median 549 ng/l; IQR 371–1,205) compared with controls (*n* = 24) (497 ng/l; 345–989) (*p* = 0.025) (Fig. 1a). This result was depending on only two patients with higher NFL concentrations compared with their matched controls. One of them was a 34-year-old woman with SLE with involvement of heart, lungs and kidneys. There was no evidence of CNS involvement of her SLE but this cannot be ruled out. The other patient with increased NFL concentrations was an 85-year old man with prostate cancer, classified as moderately differentiated carcinoma, but otherwise reported to have an active physical and social daily life. No differences in concentrations of GFAP nor S-100B, indicating astroglial damage, were shown in the patients with VZV encephalitis compared with controls (Fig. 1b,c). The median value of GFAP was 390 ng/l (IQR 324–536) for the patients with encephalitis and 279 ng/l (211–403) for the controls (Fig. 1b) and the median value of S100 was 0.66 μg/l (0.52–0.86) for the patients with encephalitis and 0.56 μg/l (0.45–0.71) for the controls (Fig. 1c). No alterations in concentrations of the AD biomarkers Aβ40, Aβ42, t-tau, p-tau nor in Aβ42/Aβ40 ratios were detected in the patients with VZV encephalitis compared with controls (Fig. 1d–g). The median value of Aβ40 was 8210 ng/l (IQR 7,480–12,404) for the patients with encephalitis and 10,247 ng/l (7,630–13,630) for the controls (Fig. 1d). The median value of Aβ42 was 710 ng/l (634–1,096) for the patients with encephalitis and 786 ng/l (614–1,105) for the controls (Fig. 1e). The median value of p-tau was 30 ng/l (20–40) for the patients with encephalitis and 30 ng/l (24–36) for the controls (Fig. 1f). The median value of t-tau was 214 ng/l (140–455) for the patients with encephalitis and 206 ng/l (156–267) for the controls (Fig. 1g). Analysed as a group, the concentrations of NFL, GFAP, S-100B, Aβ40, Aβ42, t-tau and p-tau in the CSF of the controls, were all within the reference values established in the Clinical Neurochemistry Laboratory in Mölndal, Sweden, i.e., for CSF NFL < 380 ng/l (< 30 years), < 560 ng/l (30–39 years), < 890 ng/l (40–59 years), < 1850 ng/l (> 59 years); for CSF GFAP < 750 ng/l (20–60 years), < 1250 ng/l (> 60 years); for CSF S100B < 1.7 μg/l (> 20 years); for CSF Aβ42 > 620 ng/l; for CSF Aβ42/40 ratio > 0.061; for CSF t-tau < 360 ng/l (20–50 years), < 479 ng/l (> 50 years; and for CSF p-tau < 61 ng/l^21^. In addition, no correlations of biomarker concentrations and neuropsychological test results were shown.

### Neuropsychological tests

**Figure 2:**
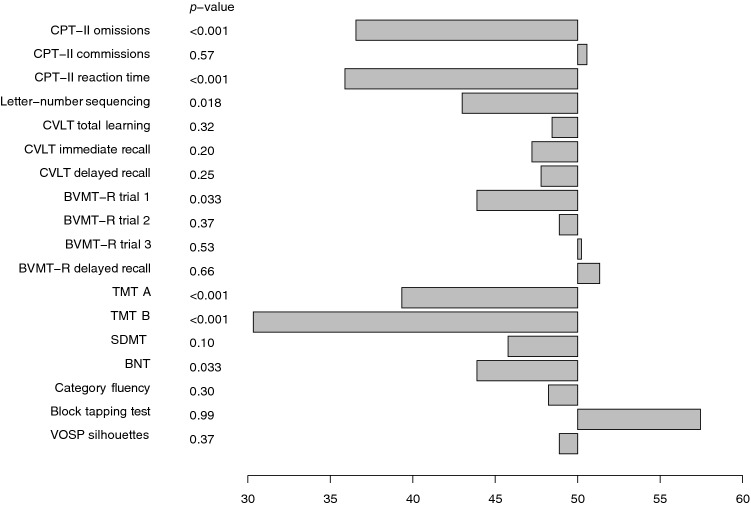
Neuropsychological test results for the total study group, presented as T-scores. P-values indicate difference between study group and normative scores.

**Figure 3:**
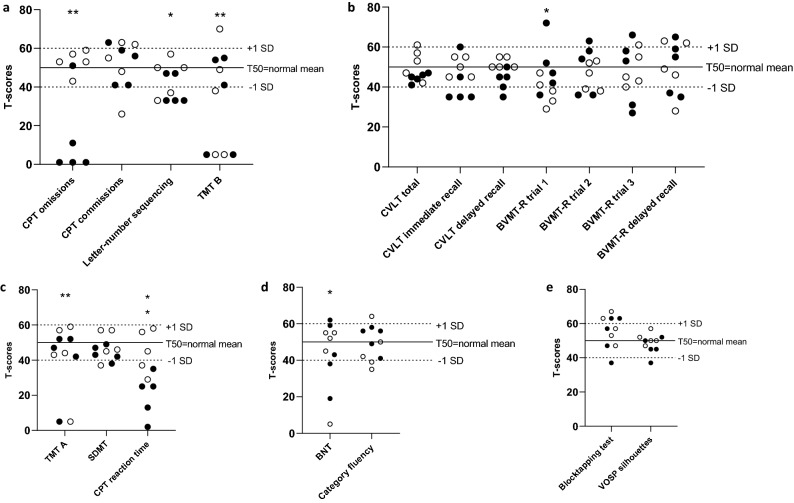
(**a–e**) Neuropsychological test results within five cognitive domains (**a**) executive functions, (**b**) learning and memory, (**c**) speed and attention, (**d**) language, (**e**) visuospatial functions, plotted as individual T-scores.  oper circle = younger ages (24; 28; 32; 34; 35 years); filled circle = older ages (52, 74, 76, 82, 85 years). Significant group differences compared to normal data (Fig. 2) are indicated by * (*p* < 0.05) or ** (*p* < 0.001). *BNT* Boston naming test, *BVMT-R* Brief visuospatial memory test revised, *Cat.* Category, *CPT* continuous performance test, *CVLT* California verbal learning test, *SD* standard deviation, *SDMT* Symbol digit modalities test, *seq.* sequencing; *TMT* Trail making test, *VOSP* Visual object and space perception.

The patients with VZV CNS infection performed significantly worse (*p* < 0.05) compared with the normal mean scores on seven out of 18 analysed neuropsychological test variables (Fig. 2): on 3 out of 4 test variables in the executive function domain: CPT omissions (*p* < 0.001), letter-number sequencing (*p* = 0.018), TMT B (*p* < 0.001); 2 out of 3 test variables in the speed/attention domain: CPT hit reaction time (*p* < 0.001), TMT A (*p* < 0.001); 1 out of 7 test variables in the learning/memory domain: BVMT-R trial 1 (*p* = 0.033); and 1 out of 2 test variables in the language domain:; BNT (*p* = 0.033). There were no significant differences within the visuospatial domain. Individual test scores for each participant are plotted in Fig. 3.

The neuropsychological test results were compared with normative data from different previously published sources that were already adjusted for age and education. However, due to the varying ages in our study participants, we also checked for associations between the participants’ T-scores and age. Older individuals performed worse in comparison with their age peers compared to younger individuals on three neuropsychological tests: CPT omissions (r = -0.95; *p* < 0.001), CPT reaction time (r = -0.74, *p* = 0.01) and CVLT delayed recall (r = -0.72, *p* = 0.02). On the BVMT-R trial 1, older individuals performed slightly better compared to younger individuals (r = 0.65, *p* = 0.04).

## Discussion

This is one of few long-term studies evaluating cognitive impairment in patients with VZV encephalitis and also including investigations of associations between cognitive impairment and CSF biomarkers. We found that the patients with VZV encephalitis had signs of impairment in the domains of *executive functions*, *speed and attention*, *learning and memory* and *language* indicated by a significantly poorer performance on seven different neuropsychological test variables within these four domains. The most significant impairments were shown in the domains of *executive functions* and *speed and attention,* while a less pronounced impairment was shown in the domains of *learning and memory* and *language*. Importantly, these impairments were not accompanied by biomarker evidence of ongoing neuronal or astrocytic injury/activation or induction of AD-related brain pathologies (increased p-tau or decreased Aβ42/Aβ40 ratio) by the infection.

The results of the neuropsychological assessment are in line with two other follow-up studies^5,7^. The first one was a study of fourteen patients with different VZV CNS manifestations^7^ and the second one included nine patients with VZV encephalitis^5^. In both these studies, signs of cognitive impairment were shown in the domains of speed and attention and learning and memory. In the most recent study of fourteen patients^7^, cognitive impairment was also reported in the domain of executive functions, as in our study. However, a third study of eight patients with VZV encephalitis revealed poorer performance in only one test in the domain of visuospatial function^6^. Yet, all conducted studies include a small number of participants, and in the second and third study, only few patients were followed-up after 1 year, making the results difficult to interpret. Nevertheless, our results strengthen the notion that patients with VZV CNS infection may be impaired in several cognitive domains a long time after acute disease and the impairment seems to predominantly include the domains of *executive functions* and *speed and attention,* and to some extent *learning and memory and language*.

As cognitive impairment may be associated to altered concentrations of CSF biomarkers, we aimed to investigate this relation. NFL concentrations were increased in the patients with VZV encephalitis compared with controls. However, this result was depending on only two patients of whom at least one of them had a disease that may involve CNS, although this 34-year-old woman had no evidence of CNS involvement of her SLE. The other patient was an 85-year-old man with prostate adenocarcinoma where CNS involvement is rare but do occur. Hence, we do not interpret this increase in CSF NFL as evidence of an association to VZV, even though it cannot be ruled out. Increased concentrations of NFL and GFAP have previously been reported in patients with VZV CNS infection^10,11^ and were most pronounced in VZV encephalitis. Similar findings have been reported in patients with other CNS infections, such as HSE, tick-borne encephalitis (TBE) and neuroborreliosis^22,23^. However, in these studies the CSF biomarkers were only measured up to the first few months after acute disease and the increased levels were presumably corresponding to the acute CNS damage. If virus is cleared from CNS with the help of antivirals, one could expect that no ongoing neuronal or astroglial damage occur. In autopsy reports VZV is only rarely detected in brain tissue, in contrast to herpes simplex type-1(HSV-1)^24^.

The other CSF biomarkers investigated in this study, Aβ40, Aβ42, t-tau and p-tau, are most commonly used to characterize AD and other dementias. We could not detect any alterations in these biomarkers long time after acute disease that would indicate a connection between VZV to AD or other dementias. Nevertheless, as previously mentioned, patients with herpes zoster have been shown in recent large epidemiological studies to run a greater risk of developing dementia, in particular AD^8,9^. A suggested mechanism for this link, although not supported by our results, is increased toxic amyloid burden by extracellular aggregation of cellular peptides into amyloid fibrils mediated by VZV glycoprotein B peptides^13^. Among other CNS infections, patients with HSE have expressed an AD-like biomarker pattern with regard to increased CSF t-tau, p-tau and decreased Aβ42^15^. Several authors have proposed a connection between HSV-1 and development of AD^15,25,26^. However, this AD-like biomarker pattern may also be an expression of the direct cytotoxic effect that HSV-1 exerts on neurons with inflammation and focal necrosis of brain tissue merely at acute disease^27^.

Despite the lack of altered concentrations of specific CSF biomarkers there may still exist an increased vulnerability of developing dementia as mild cognitive impairment is one of the risk factors^28^. Both AD and other forms of dementia is preceded by mild cognitive impairment. If biomarker alterations are lacking, other dementia forms than AD, e.g. vascular dementia, are more likely the cause of mild cognitive impairment^28,29^. VZV encephalitis is suggested to be primarily a vasculopathy with vessel wall infection^30^ and in the context of our results a progression to dementia may not necessarily be in the form of AD. However, further studies are needed to investigate the association of VZV to AD and other dementias.

Lastly, several cognitive abilities are negatively affected by aging, such as processing speed and abilities within the memory, language, visuospatial, and executive function domains^31^. The participants in our study were of varying ages, and their test scores were analysed as T-scores in relation to normative age- and education adjusted data. However, we found a significant negative correlation with age for five neuropsychological test variables. Especially, older study participants had lower T-scores on CPT omissions (failure to respond when asked to respond on certain letters on a screen) and CPT reaction time. These results indicate that the older individuals performed worse on these measures of processing speed and attention compared to age peers, than the younger study participants whose results were largely on par with expected levels for age peers. Possibly, these results could be related to cognitive difficulties caused by factors other than VZV infection in our older individuals, and the number of participants is too small to make firm conclusions. If replicated, the results could suggest that VZV infection lead to more impairment in the speed/attention cognitive domain in older individuals compared to in younger individuals.

There are some limitations of this study, such as small number of participants and no CSF biomarker data or neuropsychological assessment at the time for acute disease. In addition, this study was conducted without controls for the neuropsychological assessment. Previously published age- and education adjusted normative data were used instead.

In conclusion, our study demonstrates that patients with VZV encephalitis suffer from cognitive impairment in the domains of *executive functions* and *speed and attention,* and to some extent *learning and memory and language,* several years after acute disease. However, no convincing evidence were shown of alterations in the CSF biomarkers GFAP, NFL, S-100B, Aβ40, Aβ42, t-tau or p-tau indicating an association to ongoing neuronal damage or dementia.

### Ethical approval

The Medical Ethics Committee at Gothenburg University approved the study (EPN 229–14) and informed consent was obtained from all patients. The study has been performed in accordance with the ethical standards laid down in the 1964 Declaration of Helsinki and its later amendments.

## Data Availability

The datasets used and/or analysed during the current study are available from the corresponding author on reasonable request.

## References

[1] Persson A, Bergstrom T, Lindh M, Namvar L, Studahl M (2009). Varicella-zoster virus CNS disease–viral load, clinical manifestations and sequels. J. Clin. Virol. Off. Publ. Pan Am. Soc. Clin. Virol..

[2] Grahn A, Nilsson S, Nordlund A, Linden T, Studahl M (2013). Cognitive impairment 3 years after neurological Varicella-zoster virus infection: a long-term case control study. J. Neurol..

[3] Mailles A (2012). Long-term outcome of patients presenting with acute infectious encephalitis of various causes in France. Clin. Infect. Dis. Off. Publ. Infect. Dis. Soc. Am..

[4] Granerod J (2010). Causes of encephalitis and differences in their clinical presentations in England: a multicentre, population-based prospective study. Lancet. Infect. Dis.

[5] Hokkanen L (1997). Subcortical type cognitive impairment in herpes zoster encephalitis. J. Neurol..

[6] Wetzel K (2002). Good cognitive outcome of patients with herpes zoster encephalitis: a follow-up study. J. Neurol..

[7] Grahn A, Nilsson S, Nordlund A, Linden T, Studahl M (2013). Cognitive impairment 3 years after neurological Varicella-zoster virus infection: a long-term case control study. J. Neurol..

[8] Chen VC (2018). Herpes zoster and dementia: a nationwide population-based cohort study. J. Clin. Psychiatry.

[9] Tsai MC (2017). Increased risk of dementia following herpes zoster ophthalmicus. PLoS ONE.

[10] Grahn A (2013). Cerebrospinal fluid biomarkers in patients with varicella-zoster virus CNS infections. J. Neurol..

[11] Lindstrom J, Grahn A, Zetterberg H, Studahl M (2016). Cerebrospinal fluid viral load and biomarkers of neuronal and glial cells in Ramsay Hunt syndrome. Eur. J. Neurosci..

[12] Blennow K, Zetterberg H (2018). Biomarkers for Alzheimer's disease: current status and prospects for the future. J. Intern. Med..

[13] Bubak AN (2019). Varicella zoster virus infection of primary human spinal astrocytes produces intracellular amylin, amyloid-beta, and an amyloidogenic extracellular environment. J. Infect. Dis..

[14] Gisslen M (2009). Amyloid and tau cerebrospinal fluid biomarkers in HIV infection. BMC Neurol..

[15] Krut JJ (2013). Cerebrospinal fluid Alzheimer's biomarker profiles in CNS infections. J. Neurol..

[16] Nathan PJ (2017). Association between CSF biomarkers, hippocampal volume and cognitive function in patients with amnestic mild cognitive impairment (MCI). Neurobiol. Aging.

[17] Han SD (2012). Beta amyloid, tau, neuroimaging, and cognition: sequence modeling of biomarkers for Alzheimer's Disease. Brain Imaging Behav..

[18] Gaetani L (2019). Cerebrospinal fluid neurofilament light chain tracks cognitive impairment in multiple sclerosis. J. Neurol..

[19] Gaetani L (2018). A new enzyme-linked immunosorbent assay for neurofilament light in cerebrospinal fluid: analytical validation and clinical evaluation. Alzheimer's Res. Ther..

[20] Rosengren LE (1992). A sensitive ELISA for glial fibrillary acidic protein: application in CSF of children. J. Neurosci. Methods.

[21] Alcolea D (2019). Agreement of amyloid PET and CSF biomarkers for Alzheimer's disease on Lumipulse. Ann. Clin. Transl. Neurol..

[22] Dotevall L, Hagberg L, Karlsson JE, Rosengren LE (1999). Astroglial and neuronal proteins in cerebrospinal fluid as markers of CNS involvement in Lyme neuroborreliosis. Eur. J. Neurol. Off. J. Eur. Feder. Neurol. Soc..

[23] Studahl M, Rosengren L, Gunther G, Hagberg L (2000). Difference in pathogenesis between herpes simplex virus type 1 encephalitis and tick-borne encephalitis demonstrated by means of cerebrospinal fluid markers of glial and neuronal destruction. J. Neurol..

[24] Furuta Y (1992). Latent herpes simplex virus type 1 in human geniculate ganglia. Acta Neuropathol..

[25] Eimer WA (2018). Alzheimer's disease-associated beta-amyloid is rapidly seeded by herpesviridae to protect against brain infection. Neuron.

[26] Itzhaki RF, Wozniak MA (2008). Herpes simplex virus type 1 in Alzheimer's disease: the enemy within. J. Alzheimer's Dis. JAD.

[27] Esiri MM (1982). Herpes simplex encephalitis. An immunohistological study of the distribution of viral antigen within the brain. J. Neurol. Sci..

[28] Petersen RC (2014). Mild cognitive impairment: a concept in evolution. J. Intern. Med..

[29] Mattsson N (2009). CSF biomarkers and incipient Alzheimer disease in patients with mild cognitive impairment. JAMA J. Am. Med. Assoc..

[30] Gilden D, Cohrs RJ, Mahalingam R, Nagel MA (2009). Varicella zoster virus vasculopathies: diverse clinical manifestations, laboratory features, pathogenesis, and treatment. Lancet Neurol..

[31] Harada CN, Natelson Love MC, Triebel KL (2013). Normal cognitive aging. Clin. Geriatr. Med..

[32] Conners CK, M. S. *Conners' Continuous Performance Test II: Computer Program for Windows Technical Guide and Software Manual* (Multi-Health Systems, 2000).

[33] D, W. *Wechsler Memory Scale-Third Edition manual, Swedish version.*, (Harcourt Assessment, NCS Pearson Inc / Katarina Tryck AB, 2008).

[34] Tombaugh TN (2004). Trail Making Test A and B: normative data stratified by age and education. Arch. Clin. Neuropsychol..

[35] Delis DC, K. J., Kaplan E, Ober BA. *California Verbal Learning Test - second edition. Adult version. Manual* (Psychological Corporation, 2000).

[36] RHB, B. *Brief visuospatial memory test - revised: Professional manual* (Psychological Assessment Resources, Inc, 1997).

[37] Sheridan LK (2006). Normative symbol digit modalities test performance in a community-based sample. Arch. Clin. Neuropsychol..

[38] Tallberg IM (2005). The Boston naming test in Swedish: normative data. Brain Lang..

[39] Tallberg IM, Ivachova E, Jones Tinghag K, Ostberg P (2008). Swedish norms for word fluency tests: FAS, animals and verbs. Scand. J. Psychol..

[40] Warrington EK, J. A. *The Visual Object and Space Perception Battery.* . (Thames Valley Test Company, 1991).

